# Theoretical optimization of the removal of cryoprotective agents using a dilution-filtration system

**DOI:** 10.1186/1475-925X-13-120

**Published:** 2014-08-21

**Authors:** Heyuan Qiao, Weiping Ding, Sijie Sun, Liangquan Gong, Dayong Gao

**Affiliations:** Center for Biomedical Engineering, University of Science and Technology of China, Hefei, Anhui 230027 China; Department of Electronic Science and Technology, University of Science and Technology of China, Hefei, Anhui 230027 China; Department of Bioengineering, University of Washington, Seattle, WA 98195 USA; Department of Mechanical Engineering, University of Washington, Seattle, WA 98195 USA

**Keywords:** Mass transfer, Cryoprotective agent, Osmotic damage, Red blood cell

## Abstract

**Background:**

In the cryopreservation of blood, removing cryoprotectants from the cryopreserved blood safely and effectively is always being focused on. In our previous work, a dilution-filtration system was proposed to achieve the efficient clearance of cryoprotectants from the cryopreserved blood.

**Method:**

In this study, a theoretical method is presented to optimize the diluent flow rate in the system to further reduce the osmotic damage to red blood cells (RBCs) and shorten the washing time necessary to remove cryoprotective agents (CPAs), based on a discrete mass transfer concept. In the method, the diluent flow rate is automatically adjusted by a program code in each cycle to maximize the clearance of CPAs, whereas the volume of RBCs is always maintained below the upper volume tolerance limit.

**Results:**

The results show that the optimized diluent flow rate can significantly decrease the washing time of CPAs. The washing time under the optimized diluent flow rate can be reduced by over 50%, compared to the one under the fixed diluent flow rate. In addition, the advantage of our method becomes more significant when the blood flow rate is lower, the dilution region volume is larger, the initial CPA concentration is higher, or the cell-swelling limit set by the system is smaller.

**Conclusion:**

The proposed method for the dilution-filtration system is an ideal solution for not only guaranteeing the volume safety of RBCs but also shortening the washing time of CPAs. In practice, the optimization strategies provided here will be useful in the rapid preparation of cryopreserved blood for clinical use.

## Introduction

Cryoprotective agents (CPAs) are widely used in the cryopreservation of blood to protect red blood cells (RBCs) from cryoinjury [1–3]. However, due to their negative effects to the human body (e.g. DMSO is considered to be toxic, glycerol is responsible for the osmotic lysis of cells when the glycerolized RBCs are directly transfused), CPAs must be removed before clinical transfusion to patients [4–7]. As one of the key steps before the clinical use of cryopreserved blood, the process of removing CPAs might result in osmotic damage to RBCs and thus a functional decrease of RBCs. Consequently, this area has been a focus of research [8–10].

In practice, there are two major criteria to weigh a method for removing CPAs: one is the osmotic damage to RBCs (or the recovery rate of RBCs) and the other is the washing time or the removal efficiency of the CPAs. A high recovery rate of RBCs can improve the treatment, and a short washing time can significantly reduce the waiting time of patients, especially during emergency treatment. To remove CPAs from cryopreserved blood safely and effectively, various methods have been proposed over the past decades. During early stages, a single-step centrifugation method was applied where cryopreserved blood was diluted in an isotonic saline solution and then centrifuged to remove the supernatant containing CPAs. This method is simple and efficient for removing CPAs but causes serious osmotic damage to RBCs. Alternatively, a multi-step centrifugation method was designed to reduce the osmotic damage to RBCs [8, 10–13]. In the literature, many efforts focus on its improvement and optimization, from the Fixed Volume Steps (FVS) [14] to the Fixed Molarity Steps (FMS) [15] until the Fixed level of Shrinkage/Swelling steps (FSS) [16–18]. Recently, Lusianti et al. even reduced the deglycerolization time to several minutes [10]. In the multi-step centrifugation method, although many achievements are reached, the complex operation to some extent is still inconvenient in practice, especially for emergency use. To avoid potential cell clumping and loss caused by the centrifugation, a dialysis-based method that was originally used in blood purification was proposed to remove CPAs [19, 20]. In the method, the blood containing CPAs flows inside hollow fibers whereas the isotonic solution flows outside countercurrently. As the CPAs inside hollow fibers but outside RBCs are gradually transferred out of hollow fibers along the blood flow direction, the dialysis-based method provides a friendly environment with the stepwise decreasing CPA concentration for RBCs. This method is relatively simple to operate and can reduce osmotic damage to RBCs by easily controlling the flow conditions; however, the efficiency of the procedure is restricted by the mass transfer rate of the CPAs across the fiber membrane.

**Figure 1:**
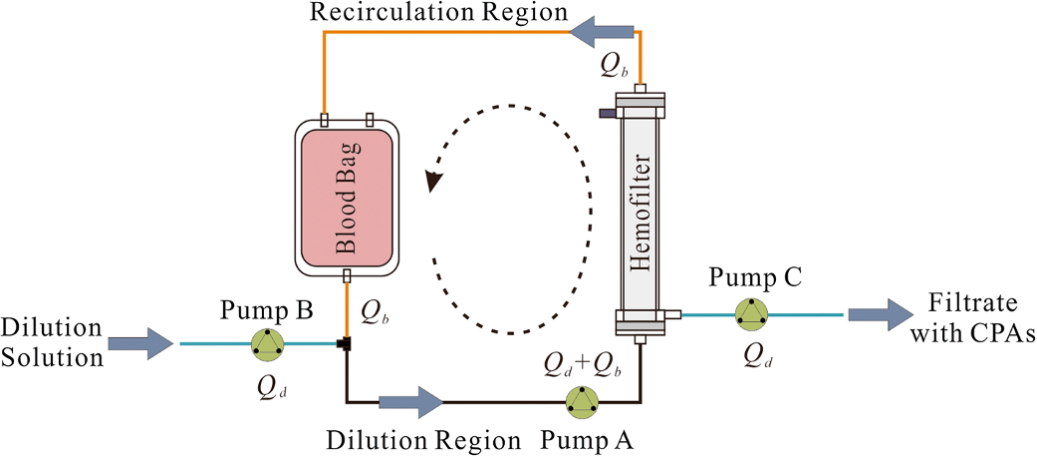
**Schematic of the dilution-filtration system.**

In our previous work, a novel system, based on the literature [21], named dilution-filtration and shown in Figure 1, was developed [22]. In the system, the blood containing CPAs is circulated in a closed-loop system and is submitted to continuous dilution and filtration processes. As RBCs are concentrated or enriched by a hemofilter, the solution outside the cells containing the CPAs is removed. Based on our preliminary results from that study, basic guidance was provided to alleviate the osmotic damage to RBCs and shorten the washing time necessary to remove CPAs by adjusting the blood and/or diluent flow rates [22]; however, those common measures sometimes are not sufficiently effective, especially in shortening the washing time.

Therefore, in this work, a method is proposed to optimize the dilution-filtration system to further reduce the osmotic damage to RBCs and shorten the washing time necessary to remove CPAs. The optimization strategy presented here remarkably improves the dilution-filtration system and makes it more appropriate for clinical use.

## Methods

### Mass transfer equation revisited

In the dilution-filtration system, the volume variation of RBCs and the concentration change of CPAs are influenced by many factors, e.g., the volume of blood, the hematocrit in the blood, the flow rate of the blood, the flow rate of the dilution solution, the performance of the hemofilter, and the parameter of the tubing (Figure 1). To simplify the issue, based on our preliminary results [22, 23], we focused on the flow rate and tubing volume. We assumed that the volume of the blood to be washed is small (i.e., no blood is left in the blood bag during operation), and thus, the blood bag serves as a reservoir only at the beginning and then is considered as a tube starting from the second cycle. Under this assumption, the mixing complexity of the RBCs in a blood bag can be neglected and the behaviors of all cells can be assumed the same. In the system, cells go through three major steps: dilution (black line), filtration (hemofilter) and recirculation (red line and blood bag), the blood flow rate (*Q*_*b*_) and the diluent flow rate (*Q*_*d*_) can be controlled by system software, and the tubing volume can be designed before operation.

**Figure 2:**
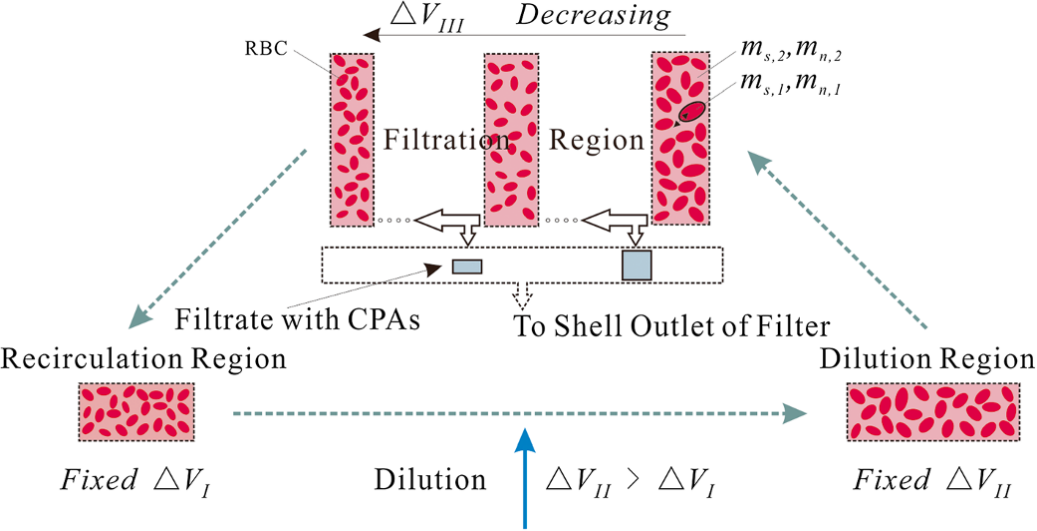
**Schematic of unit volumes in different regions.**

To monitor the behavior of individual cells, a discrete method that was developed in our previous work is used here [20]. In brief, we assumed that blood and diluent could be divided into limited and unlimited numbers of tiny units, respectively. With this division, the flow space in the system (including the tubing and the hemofilter) is also divided into a limited number of compartments of different lengths. Thus, the flow in the system can be simulated by the unit shift from one compartment to the next (the blood unit velocity was assumed to be dependent on the blood flow rate), and the mass transfer of CPAs across the cell membrane can be approximately calculated in only one unit. By doing so, the statistical analysis of the osmotic damage to RBCs becomes possible [24]. During the shift of blood units, the unit volume is enlarged in the dilution region, reduced in the hemofilter because of filtration and restored in the recirculation region (Figure 2). CPAs inside RBCs are transported through the cell membrane, filtered out of the hemofilter and removed along with the filtrate.

In this work, the volume of the RBCs and the intracellular concentration of the CPAs were calculated by the classic two-parameter model [25, 26] and the extracellular concentration of the CPAs was obtained according to the mass conservation [23]:
12345

where m_s,1_ and m_s,2_ are CPA concentrations inside and outside of RBCs, respectively (mol/kg H_2_O). *m*_*n*,1_ and *m*_*n*,2_ are intracellular and extracellular NaCl concentrations, respectively (mol/kg H_2_O); *V*_*s*,1_ and *V*_*s*,2_ are CPA volumes inside and outside of RBCs, respectively (μm^3^); *L*_*p*,*c*_ (m/Pa/s), and *P*_*s*,*c*_ (m/s) are hydraulic permeability and solute permeability of the RBC membrane, respectively. *V*_*c*_ is the RBC volume (μm^3^); *A*_*c*_ is the cell membrane area (μm^2^); *R* is the universal gas constant (J/mol/K); T is the absolute temperature (K). *V*_*bc*_ refers to the osmotically inactive volume of the cells at the isotonic condition (μm^3^);  is the CPA partial molar volume (l/mol); *α* is the cell density; *ΔV* is the volume of blood units. Here, it should be noted that *ΔV* is a variable in the hemofilter but keeps constant in the tubing [23] and the superscript *0* denotes the previous time.

Because of the dilution, the extracellular CPA and NaCl concentrations will change at the dilution point (Figure 1). Based on mass conservation, the concentration changes can be described by the following equations:
67

where superscripts I and II denote blood units before and after the dilution point, respectively, and *d* denotes the NaCl concentration in the diluent.

In the simulation, a solution with NaCl and glycerin (80 mL) and a solution with only NaCl were used to mimic blood and diluent, respectively. The initial NaCl and glycerin concentrations were 290 and 6500 mOsm, respectively. The blood hematocrit level was set at 30%. The plasma filter (PlasmfloTM AP-05H/L, ASAHI Co., Japan) was used (the lumen volume of the filter was 85 mL). The diameter of the tubing was 4 mm. The volumes of the tubing for the dilution and recirculation regions were 5 mL and 10 mL, respectively [22]. Other parameters used in this work are shown in Table 1. In the washing process, the blood flow rate jumps at the dilution point, stays the same in the dilution region, decreases in the hemofilter, and remains unchanged in the recirculation region. We used *ΔV/Δt* to represent the blood flow rate and *Δt* was set to a given constant; as a result, the blood unit volume *ΔV* also changed along the blood flow direction [23].

**Table 1:** **Parameters used in this paper**

Parameter	Parameters definition	Units	Values	Reference
*L*_*p*,*c*_	Hydraulic permeability	m/(Pa·s)	1.74 × 10^-12^	[27]
*P*_*s*,*c*_	Glycerol permeability	m/s	6.61 × 10^-8^	[27]
*A*_*c*_	RBC surface area	μm^2^	134.1	[28]
*V*_*iso*_	RBC volume at isotonic conditions	μm^3^	89.8	[28]
*V*_*bc*_	Osmotically inactive cell volume	μm^3^	39.2	[28]
*T*	Absolute temperature	K	298.15	

### Optimization strategy

In the optimization, our objective is to find a series of optimal diluent flow rates for the entire washing process when the blood flow rate and the dilution region volume are fixed. In this work, the diluent flow rate was automatically controlled by a program code that we wrote (the filtrate flow rate was synchronously controlled and was always equal to the diluent flow rate). Its value is different during different cycles but always satisfies the following two conditions: the first is that it allows cells to expand to the upper volume tolerance limit if applicable; and the second is that its value is smaller than the threshold value that the pump can reach. Under these two restrictions, an optimized diluent flow rate can be obtained for each cycle under a given blood flow rate. In the process of searching for an optimized diluent flow rate for each cycle, at the beginning, the diluent flow rate was always set to the maximal value that the system could reach, and the volume change of the RBCs was then simulated. If the peak volume was larger than the upper volume tolerance limit, the diluent flow rate was decreased by a certain small amount (0.1 mL/min), and the process was repeated until the peak volume was smaller than but near the upper volume tolerance limit. By doing so, a series of optimized diluent flow rates could be obtained for the entire washing process.

## Results and discussion

### Variation of cell volumes under fixed diluent flow rates

**Figure 3:**
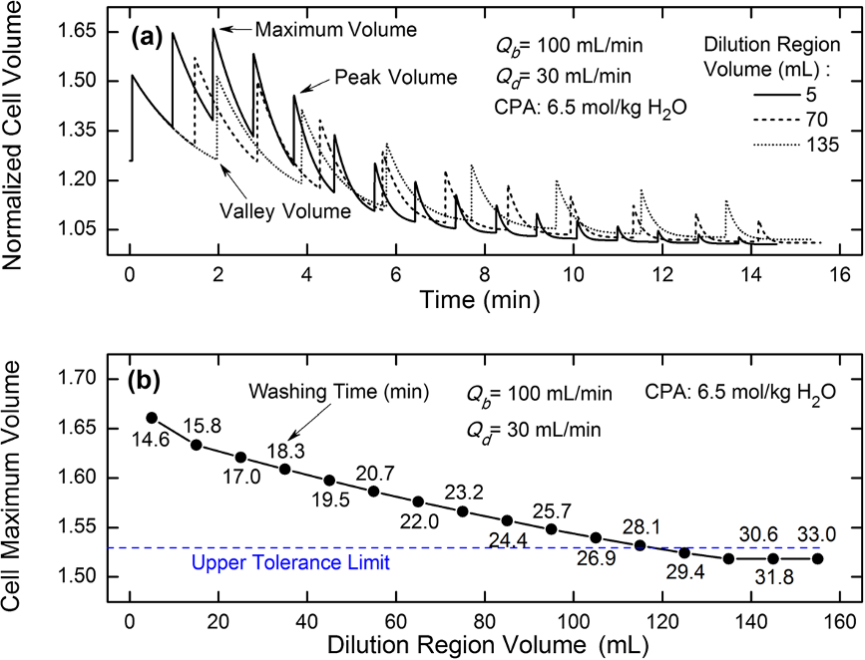
**Effects of dilution region volumes on RBC volume variation (a), RBC maximum volume and CPA washing time (b) under given blood and diluent flow rates.**

In the dilution-filtration system, the volume of the RBCs is periodically expanded and shrunk during the process of removing CPAs (Figure 3a). For a fixed diluent flow rate, the final cell volume in the previous cycle is the initial cell volume for the subsequent cycle. Therefore, if the residence time in the previous cycle is not long enough for the complete recovery of the cells (i.e., for the cells to reach a new equilibrium), the cell volume increase at the beginning of the process will accumulated. As a result, the maximum volume of the RBCs and the osmotic damage to the RBCs are increased (the maximum volume is the maximal value among all peaks in the cycles). In fact, because of the repeated swelling or shrinkage, the RBCs may suffer the sub-hemolytic injury, such as potassium leakage [29, 30], cytoskeleton damage and membrane phospholipid reorganization [31, 32]. Here, we assumed that the osmotic damage to cells is only due to the volume of cells over the upper volume tolerance limit.

In practice, the dilution region extension (i.e. the increase in the tubing length and/or diameter of the dilution region) can be used to prolong the residence time of cells. Our results show that as the dilution region volume is increased, starting from the 2^nd^ cycle, the starting volume of the RBCs is decreased; thus, the accumulation effect is alleviated and the maximum volume of the RBCs is decreased (the dot line in Figure 3a). However, it is important to note here that the utility of the dilution region extension is limited to some extent, especially when the dilution region volume is very large. Under this situation, the accumulation effect is no longer obvious, and the maximal volume of the RBCs is similar to the peak volume of the 1^st^ cycle and is nearly constant, as shown in Figure 3b. In addition, due to the increase in the residence time, the washing time necessary to remove CPAs increases almost linearly (Figure 3b). Therefore, the dilution region extension is not good enough to optimize the dilution-filtration system (here, the shear stress damage to the RBCs as a result of changes in the tubing diameter or length [33] was assumed to be neglectable).

Here, the fixed diluent flow rate is an allowable value, which makes the maximum volume of the RBCs be very close to the upper tolerance limit, and is kept constant in all cycles. In practice, the fixed diluent flow rate varies with blood flow rates and/or cryoprotectant concentrations. If the diluent flow rate is always constant, the maximum volume of the RBCs decreases as the blood flow rate increases [23]. Therefore, the fixed diluent flow rate increases with increasing blood flow rate. In this simulation, the upper tolerance limit for completely avoiding the hypotonic damage was set to 138 μm^3^, i.e., 1.53 of the isotonic volume of the RBCs according to the literature [34], and the stop condition was the CPA concentration inside the cells reaching below 5% of the initial value.

### Variation of optimized diluent flow rates

**Figure 4:**
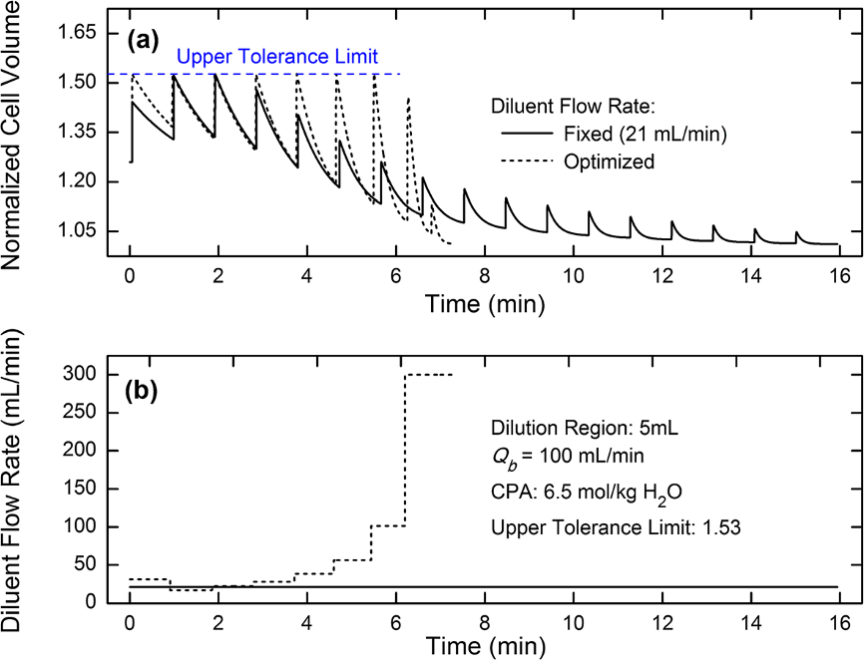
**Comparisons of cell volume variations (a) and diluent flow rates (b) between fixed and optimized conditions.**

**Figure 5:**
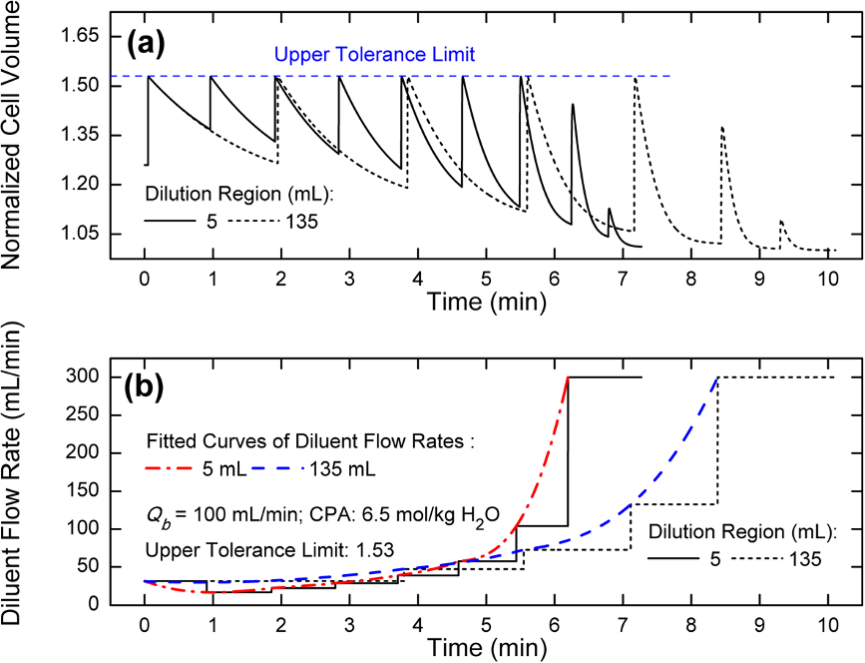
**Comparisons of cell volume variations (a) and optimized diluent flow rates (b) under different dilution region volumes.**

For a fixed diluent flow rate, due to the negative accumulation effect from the volume change of RBCs caused by the incomplete recovery of RBCs in the dilution-filtration system, the peak volume of the RBCs first increases and then decreases (Figure 3a). However, when an optimized diluent flow rate is used, the peak volume of the RBCs at the beginning is constant and then decreases rapidly (Figures 4a or 5a). During the first several cycles, the peak volume is equal to the upper tolerance limit set by the system. Due to the limit in the diluent flow rate and the low concentration of CPAs in subsequent cycles, the peak volume can no longer reach the upper tolerance limit and decreases sharply with time. In this work, the capped value of the diluent flow rate was set to 300 mL/min and we assumed that the filter used could handle the filtrate flow rate until the capped value; however, in practice, one should set the capped value according to the filter performance.

In this work, the optimized diluent flow rate was kept constant in an individual cycle whereas changed in different cycles. Our results show that it is changed step-wisely (Figure 4b). Generally, as time increases, the optimized diluent flow rate first decreases and then increases. Based on the above analysis for a fixed diluent flow rate, during the process of removing CPAs the peak volume of the RBCs first increases and then decreases (Figure 4a). Therefore, when considering the upper tolerance limit, the diluent flow rate should decrease at the beginning of the process to offset the increase in the peak volume. Consequently, a minimum flow rate exists during the variation of diluent flow rates (Figure 4b). Here, it should be pointed out that the extension of the dilution region volume can cause the increase in the washing time. However, except for the advantage that the extension can reduce the maximum volume of cells, practically, it can also make it easy to obtain the optimized diluent flow rate because of the fact that as the dilution region volume increases, the optimized diluent flow rate will gradually vary from non-monotonic to monotonic (Figure 5b).

### Comparison of washing times between fixed and optimized diluent flow rates

**Figure 6:**
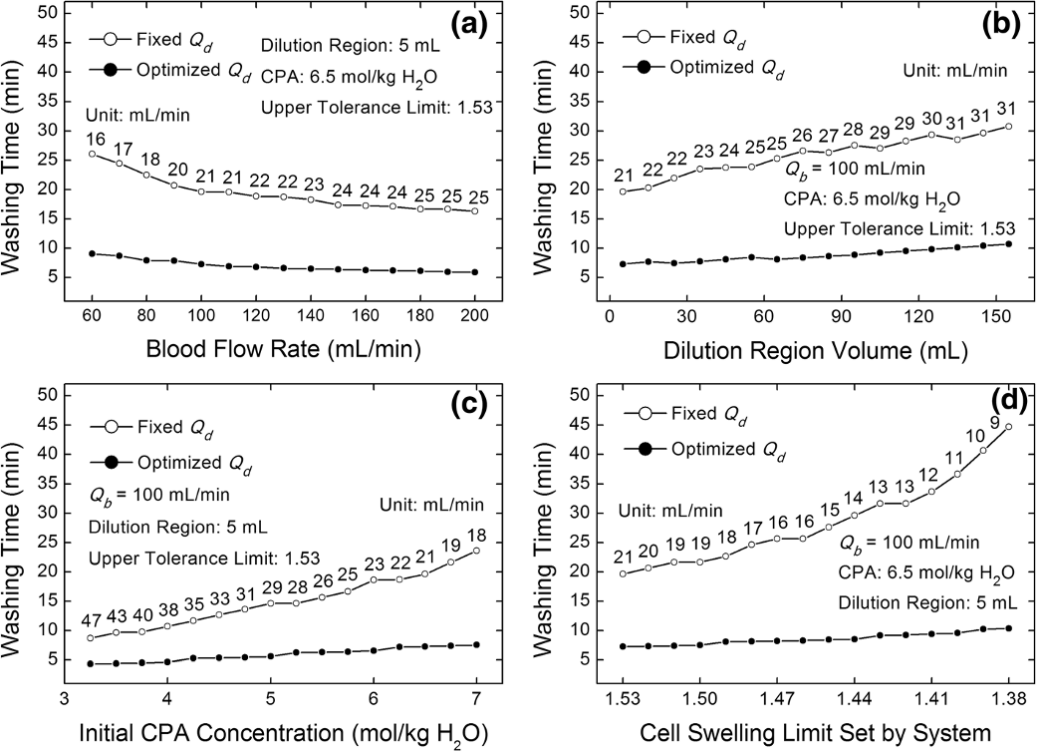
**Effects of blood flow rates (a), dilution region volumes (b), initial CPA concentrations (c), and cell swelling limits (d) on washing time under fixed and optimized diluent flow rates.**

To confirm the advantage of the optimized diluent flow rates obtained by our study, the washing times of CPAs between fixed and optimized diluent flow rates under different conditions were compared. Our results show that when an optimized diluent flow rate is used, the washing time declines dramatically (Figure 6a), and as the blood flow rate increases, the optimized diluent flow rate can still reduce the washing time by over 50%, even though the rate of decline is decreasing. In addition, as for the dilution region extension, even though it causes the decrease in the efficiency, the optimized diluent flow rate offsets this deficiency (Figure 6b). In this work, the washing time in Figure 6 undergoes a zigzag increase because, in the simulation, it is an integer multiple of the period. If the CPA concentration inside the cells is near (but still larger than) the given stop value, the system must run one more cycle to unsmooth the curve.

In the dilution-filtration system, the CPA concentration is decreased by means of dilution. Thus, for a given blood flow rate, the washing time is dominated by the diluent flow rate. When the initial CPA concentration is large, if a fixed diluent flow rate is used (it should be smaller to avoid osmotic damage to RBCs), the washing efficiency will be lowered. However, if the optimized diluent flow rate is used (i.e., the diluent flow rate in each cycle is maximized), the washing efficiency in each cycle will be maximized, and the washing time will decrease dramatically (Figure 6c). Furthermore, as the initial CPA concentration increases, the optimization becomes more remarkable. In the optimization, the choice of the cell-swelling limit is important because it greatly influences the washing time to remove CPAs. If a small cell-swelling limit is set by the system, the cells will have a small range to expand in each cycle; therefore, the diluent flow rate must be sufficiently small to cause the volume of the cells always below the limit. With a fixed diluent flow rate, a small diluent flow rate must be set due to the negative volume accumulation effect, and, as a result, the efficiency is low. However, when an optimized diluent flow rate is used, the efficiency of each cycle reaches its high limit. Consequently, the washing time decreased (Figure 6d). In addition, as the cell swelling limit decreases, a fixed diluent flow rate becomes a more significant disadvantage (the reason is that because the cell swelling limit decreases, the fixed diluent flow rate decreases sharply, consequently causing the cycle number and the washing time to increase more rapidly).

## Conclusions

The process of removing CPAs using a dilution-filtration system is theoretically optimized. In the optimization method, the diluent flow rate is designed to vary with time. The rate is adjusted automatically by a program code in the system at each cycle to maximize the clearance of CPAs, and the volume change of RBCs is always maintained below the upper volume tolerance limit. The results show that our optimization method can significantly decrease the washing time of CPAs: when the optimized diluent flow rate is used, the washing time can be reduced by over 50%. The advantage of our method becomes more remarkable when the blood flow rate is lower, the initial CPA concentration is higher, the dilution region volume is larger, or the cell-swelling limit set by the system is smaller. In addition, the extension of the dilution region volume is not a good method to optimize the dilution-filtration system as it causes the increase in the washing time of CPAs, although it sometimes can reduce the negative accumulation effect from the volume change of cells.

In this work, we describe an adaptive optimization method for the diluent flow rate in the dilution-filtration system to reduce the washing time of CPAs, based on the discrete concept [23]. In practice, one still can apply the method to the continuum mass transport concept in the literature [22] to obtain the optimized diluent flow rate. In addition, although the optimized diluent flow rate we obtain here is based on a small volume of blood, it is applicable to a large volume of blood in the literature [22, 23]. If the volume of blood to be washed is large, one only needs to extend the cycle accordingly.

As the study here is limited to a theoretical optimal solution for reducing the osmotic damage to RBCs and enhancing the speed of the removal of CPAs, we next will focus on the experimental validation of the theoretical results. In the experiments, we will monitor the concentration variation of CPAs, count the recovery rate of cells and perform the comparison between theoretical and experimental results. In addition, we will also investigate the effect of the repeated osmotic stress on the cell tolerance, the subcellular structure, and the cell permeability to validate assumptions used in this work.
